# CASES OF OPTIMALLY LOCAL SOLUTIONS TO UNMET NEEDS OF RURAL-DWELLING OLDER ADULTS: ROLES OF NESTED NETWORKS

**DOI:** 10.1093/geroni/igz038.2036

**Published:** 2019-11-08

**Authors:** Lyn Holley, Kara l Kohel, Holly Hatton-Bowers, Susan Harris-Broomfield

**Affiliations:** 1 University of Nebraska, Omaha, United States; 2 University of Nebraska–Lincoln, Lincoln, Nebraska, United States

## Abstract

Solutions developed top-down frequently make suboptimal use of resources. Programs (e.g., caregiver respite) are studied extensively; study focused on the roles of nested networks (family/locality/state/nation) that intersect in care is lacking. To identify and assess potential for improving solutions, this study examines cases acknowledged to provide optimal support. It identifies and describes network roles and intersections critical to success, with particular attention to timing and intentionality of family and community interfaces. Findings may suggest improved design and operation of programs through targeted empowerment of networks Cases were identified in cooperation with the Nebraska Extension service, and analyzed by a multidisciplinary team that included Family Science and Gerontology. Rural-dwelling older adults who benefited from the solution, expert practitioners, officials and local champions were interviewed. Analysis included private and public actors, and explains outcomes within a cultural (e.g., individualist, independent) and opportunity (e.g. information, financial and human resources) framework (Gelfand, 2003).

